# Intraindividual variations of urinary biomarkers in hospitalized children with glomerular diseases: a prospective observational study

**DOI:** 10.1007/s00431-023-05042-9

**Published:** 2023-06-10

**Authors:** Jianmei Zhou, Xuhui Zhong, Huijie Xiao, Ke Xu, Viji Nair, Maria Larkina, Wenjun Ju, Jie Ding

**Affiliations:** 1 Department of Pediatrics, Peking University First Hospital, Beijing, China; 2 Department of Internal Medicine, University of Michigan, Ann Arbor, MI USA

**Keywords:** Intraindividual variation, Biomarker, Children, Glomerular disease

## Abstract

This study aimed to assess the intraindividual variations of urinary biomarkers in hospitalized children with glomerular diseases. Hospitalized children with glomerular diseases participated in the study. For each patient, an overnight (9:00 p.m.–7:00 a.m.) urine was collected, followed by a 24-h urine (classified into four distinct periods: morning 7:00 a.m.–12:00 p.m., afternoon 12:00 p.m.–4:00 p.m., evening 4:00 p.m.–9:00 p.m., and overnight 9:00 p.m.–7:00 a.m.). The concentrations of protein, albumin, N-acetyl-beta-D-glucosaminidase, and epidermal growth factor (EGF) were measured and normalized by three correction factors (creatinine, osmolality, or specific gravity, respectively). Additionally, the 2nd overnight urine sample was grouped into different aliquots according to centrifugation, additives, storage temperature, or delayed processing. Twenty (14 boys, 6 girls) children were enrolled, with an average age of 11.3 years. Among the three correction factors, creatinine-normalized biomarkers provided the best agreements among different periods over 24 h. There were significant diurnal variations during 24 h in the concentrations of urinary protein, albumin, N-acetyl-beta-D-glucosaminidase, and EGF (*p* = 0.001, *p* = 0.003, *p* = 0.003, and *p* = 0.003, respectively). Evening urine overestimated 24-h urinary protein and albumin, while overnight urine underestimated 24-h urinary albumin. Urinary EGF showed low variability within a day or between the 2 days (coefficients of variation 10.2% and 10.6%, respectively) and excellent agreements (intraclass correlation coefficients > 0.9) with 24-h urinary concentration. Furthermore, urinary EGF was not affected by centrifugation, additives, storage temperature, or delayed processing of urine samples (all *p* > 0.05).

*  Conclusion*: Given the diurnal variations of urinary biomarkers, urine samples should be collected during the same time period in clinical practice if possible. The results also extend the evidence for urinary EGF as a relatively stable biomarker applied in the future clinical practice.

**Table:** 

**What is Known:** *• Urinary biomarkers have been widely used or discussed in making diagnoses and therapy regimens and estimating the prognosis of pediatric glomerular diseases. It remains unclear whether their levels would be affected by the time of sample collection, processing methods, and storage conditions in hospitalized children with glomerular diseases.*	
**What is New:** *• The levels of both commonly used biomarkers and novel biomarkers exhibited diurnal variations in hospitalized children with glomerular diseases.* *• Our results extend the evidence for urinary EGF as a relatively stable biomarker applied in the future clinical practice.*

**Supplementary Information:**

The online version contains supplementary material available at 10.1007/s00431-023-05042-9.

## Introduction

Urinary biomarkers have been widely used and investigated in making diagnoses and therapy regimens and estimating the prognosis of pediatric glomerular diseases. These biomarkers include both commonly used urinary biomarkers (urinary protein [1], albumin [2], and N-acetyl-beta-D-glucosaminidase [3]) and novel molecular markers (epidermal growth factor (EGF) [4–11]). Urinary protein has been acknowledged as one of the most critical risk factors [1]. Albuminuria is a marker of both glomerular and tubular damage [12], while N-acetyl-beta-D-glucosaminidase reflects the presence of proximal tubular injury [13]. Urinary EGF, as a novel and promising biomarker, is mainly derived from the synthesis and secretion of the thick ascending limb of Henle and the distal convoluted tubule [14]. These have been commonly used in clinical practice or have been widely discussed by many researchers. Therefore, it is important to detect their concentrations as accurately as possible.

However, multiple factors could cause variations in the measurements of urinary proteins [15–18]. Renal plasma flow, the glomerular filtration rate, and tubular reabsorption and/or secretion processes were previously reported to exhibit substantial circadian oscillations [19]. How circadian rhythm impacts urinary biomarkers is unclear but is receiving increasing attention in the biomarker field. Given that urinary biomarkers may be influenced by circadian rhythm, the optimal way is to measure biomarkers from the 24-h urine, which has been widely used for the quantification of proteinuria in clinical practice and clinical studies [20–22]. However, the successful 24-h urine collection involves multiple challenges, including inconvenient procedures (strict requirements for sample collection time), inaccurate collection [23], and a high likelihood of sample contamination [24]. It is especially challenging for pediatric patients, who understandably have poor compliance. To address this, spot urine collection is often used as a surrogate for 24-h urine. A previous study observed that urinary protein levels in upright (i.e., in the daytime) urine were significantly higher than that in supine (i.e., in the nighttime) in healthy children [25]. In contrast, no apparent diurnal variation of EGF was observed in healthy adults [26, 27]. Because children with glomerular diseases showed significant increase of urinary protein compared with healthy children, it is unclear whether their diurnal patterns would differ.

Furthermore, unlike the commonly used urinary proteins, novel biomarkers were generally measured after samples were cryopreserved for a period of time. It is not quite clear if the pre-analytical conditions affect the biomarker concentrations. Although a previous study reported that EGF in urine samples could remain stable after repeated freeze–thaw cycles or cryopreservation for up to 6 months [28], it remains unclear whether the levels of EGF in urine would be affected by the step of centrifugation, additives, freezing temperature, or delayed processing. Thus, the current study aimed to determine the impact of sample collection time, urine processing methods, and storage conditions on the levels of urinary biomarkers in children with glomerular diseases.

## Subjects and methods

### Subjects

Hospitalized children from August 2019 to November 2019 were prospectively enrolled in the study if they met all the following inclusion criteria: (1) age ≥ 3 years old and < 18 years old, (2) diagnosed with glomerular diseases, and (3) 24-h urinary protein > 150 mg or urinary protein:creatinine ratio (PCR) > 0.2 g/g. Patients were excluded if they met one or more criteria as follows: (1) rejected to adhere to instructions for urine sample collection and (2) patients with potential congenital, genetic, metabolic, or hepatic diseases. Patients were provided with the kidney diet prescribed by the nutritionists of the hospital. They were not allowed to leave the ward and were educated to avoid strenuous exercise, according to the routine hospital regulations for hospitalized patients in our hospital.

The study was performed in line with the principles of the Declaration of Helsinki and was approved by the Ethics Committee of Peking University First Hospital (approval no. 2019–143). Written informed consent to participate in this study was provided by participants’ parents or guardians.

### Data collection and clinical assessment

Patient demographic and clinical information during urine collection, including age, sex, height, weight, serum creatinine, cause of disease, and treatment, were obtained via electronic medical records. Before the sample collection, a paper-based self-reporting questionnaire (Supplementary file 1) was given to the patients or guardians to record patients’ time of meals and sleep during the sample collection, with the 5-min education for interpreting how to fill in the information on the questionnaire. To avoid recall bias, patients or their guardians were requested to fill in the questionnaire before each meal and sleep, respectively. Questionnaires were taken back by the assigned investigator after the sample collection was completed. Age- and sex-specific body mass index (BMI) percentile was calculated according to World Health Organization Center BMI growth charts, and obesity was assessed according to the definition of World Health Organization [29]. The updated Schwartz creatinine-based equation for children was used to calculate eGFR [30]. Patients were classified as having hypertension if their blood pressure was above the 95th percentile of age- and sex-matched Chinese healthy children [31].

### Urine collection and processing

**Fig. 1:**
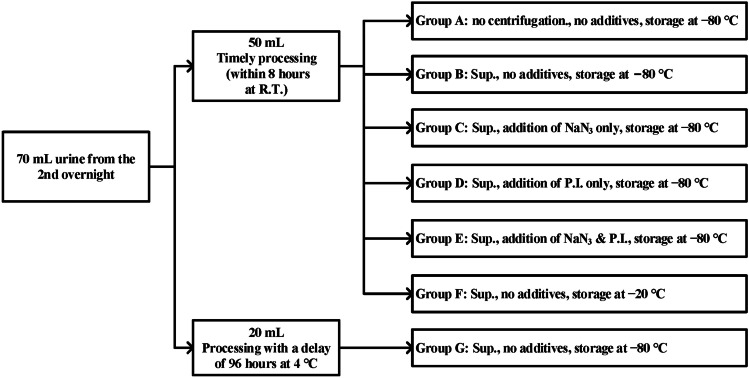
Urine processing procedure and grouping. Samples were centrifuged at 4 °C with 4000 rpm for 15 min. Vortex mixing for 15 s was performed before measurement. RT, room temperature. Sup., supernatant. NaN3, sodium azide (1.25 mM). PI, protease inhibitor (1.25 µL/mL, P1860-1ML, Sigma-Aldrich, USA)

Each patient voided spontaneously into a container and was educated to collect every urination during the process of urine collection. An overnight (9 p.m.–7 a.m.) urine was collected from each patient, followed by one 24-h urine collection (split into four distinct periods: morning period 7 a.m.–12 p.m., afternoon period 12 p.m.–4 p.m., evening period 4 p.m.– 9 p.m., and overnight period 9 p.m.–7 a.m.). The urine volume was recorded at the end of each period. The 2nd overnight urine was further divided into seven groups in terms of centrifugation, additives, frozen temperature, and delayed processing (see Fig. 1).

### Measurements

Urinary protein, albumin, and N-acetyl-beta-D-glucosaminidase levels were measured with automated biochemistry analyzers. Urinary protein was measured with a Beckman Coulter AU5800 (CA, USA) clinical chemistry analyzer using the pyrogallol red method. Albumin was measured with a Beckman Coulter IMMAGE 800 (CA, USA) clinical chemistry analyzer using a nephelometric immunoassay. N-acetyl-beta-D-glucosaminidase was measured with a Hitachi 7180 (Tokyo, Japan) clinical analyzer using the MNP-G1CNAc substrate method. Urinary EGF was measured after urine samples were cryopreserved for 3 months. Urinary EGF was measured with the Human EGF Quantikine ELISA Kit (SEG00, R&D Systems, USA) respectively. The samples were measured in duplicate. The concentrations of all the above urinary biomarkers were divided by urinary creatinine, osmolality, or (specific gravity-1) × 100 [32] for normalization of hydration status. Intra-assay variation and inter-assay variation were within 4% and 8%, respectively (evaluated by quality control, QC21, R&D Systems). The levels of urinary albumin in nine patients were excluded from data analyses for exceeding the upper limit of measurements.

### Statistical analysis

Data were analyzed with R (4.1.1). Normal distribution of the variables was tested using Shapiro–Wilk test. Normalized data were described by the mean and standard deviation. Skewed data were described by the median with interquartile range. The agreements among four periods for the levels of urinary biomarkers normalized by different correction factors were evaluated by the intraclass correlation coefficients (ICC). ICC values below 0.50 represent poor agreement, values between 0.50 and 0.75 moderate agreement, values between 0.75 and 0.90 good agreement, and values above 0.90 excellent agreement [33]. The concentrations of 24-h urinary biomarkers were calculated based on the weighted average of their corresponding concentrations of four periods, considering that urine volumes differed among the four periods. Log_2_ transformation was used to reduce the skewness. A linear mixed effects model was used to assess the diurnal variations by comparing the levels of biomarkers across four periods over 24 h, with Bonferroni corrections for multiple testing. To further explore whether the diurnal variations would differ in different subgroups, patients were stratified according to age (pre-adolescent < 12 years or adolescent ≥ 12 years), obesity (yes or no), usage of diuretics (yes or no), or blood product transfusion (yes or no), respectively. Tests of interaction were performed by linear mixed effects model. Within-day variations were assessed by the coefficients of variation (standard deviation/mean × 100%) among the four periods during 24 h. Day-to-day variations were evaluated by the coefficients of variation between the two overnight periods. The Wilcoxon signed-rank test was used for comparison between within-day variations and day-to-day variations and to assess the impact of centrifugation, additives, storage temperature, and delayed processing. The agreement between each period and 24 h was evaluated by ICC and the average ratios of urinary biomarkers at each period to those of the 24-h urine. A ratio < 0.8 was evaluated as underestimation of the 24-h urinary biomarkers, and a ratio > 1.2 was evaluated as overestimation of the 24-h urinary biomarkers [34]. A *p* < 0.05 (two-sided) was considered statistically significant.

## Results

### Patient characteristics

Twenty patients were enrolled during the study period. The flow diagram for patient enrollment was shown in Supplementary Fig. 1. The demographic and clinical characteristics of these patients are shown in Table 1. Most patients (19 out of 20 patients, 95%) had normal eGFR (> 90 ml/min/1.73 m ^2^). As hospitalized children, patients generally woke up between 5:30 a.m. and 7:00 a.m. in the morning (19 out of 20 patients, 95%), had breakfast between 7:00 a.m. and 7:30 a.m. (18 out of 20 patients, 90%), had lunch between 11:00 a.m. and 12:00 p.m. (19 out of 20 patients, 95%), had dinner between 5:00 p.m. and 5:30 p.m. (all patients, 100%), and went to sleep between 8:30 p.m. and 10:30 p.m. (18 out of 20 patients, 90%).

**Table 1:** Demographic and clinical characteristics of enrolled patients (*n* = 20)

Characteristics	Value
Age (years)	11.3 ± 4.2
Sex (boys)	14 (70%)
BMI percentile	90.0 (26.2–97.2)
Obesity	5 (25%)
Serum creatinine (μmol/l)	45.6 (36.2–58.2)
eGFR (ml/min/1.73 m^2^)	120.3 (105.4–135.1)
Protein creatinine ratio (g/g)	5.2 (1.5–10.5)
Cause of glomerular diseases	
Idiopathic nephrotic syndrome	16 (80%)
Primary IgA nephropathy	2 (10%)
Henoch-Schonlein purpura nephritis	1 (5%)
Lupus nephritis	1 (5%)
Hypertension	7 (35%)
Blood product transfusion (plasma or immunoglobulin)	7 (35%)
Usage of diuretics	4 (20%)
Usage of ACEi/ARBs	11 (55%)
Usage of steroid or immunosuppressive agents	19 (95%)

Data are reported as the mean ± standard deviation for normalized data, median (interquartile range) for skewed data, and *n* (%) for categorical variables

*BMI* body mass index. *eGFR* estimated glomerular filtration rate, *ACEi/ARBs* angiotensin-converting enzyme inhibitors/angiotensin receptor blockers

### Agreement among four periods for urinary biomarkers normalized by different correction factors

As shown in Table 2, the protein and N-acetyl-beta-D-glucosaminidase had excellent agreement among four periods when normalized by creatinine (*ICC* > 0.9) but good and poor agreement when normalized by osmolality (*ICC* 0.844 and 0.836, respectively) and specific gravity (*ICC* 0.381 and 0.426, respectively). Albumin showed moderate agreement when normalized by any of the three correction factors (*ICC* 0.595–0.707). The levels of EGF exhibited good agreement when normalized by creatinine (*ICC* = 0.893) but moderate agreement when normalized by osmolality or specific gravity (*ICC* 0.672 and 0.581, respectively).

**Table 2:** The agreement among four periods for urinary biomarkers normalized by creatinine, osmolality, and specific gravity, respectively

	ICC	95% *CI*
Protein		
Creatinine	0.901	0.804–0.958
Osmolality	0.844	0.716–0.93
Specific gravity	0.381	0.135–0.663
Albumin		
Creatinine	0.610	0.301–0.862
Osmolality	0.595	0.296–0.854
Specific gravity	0.707	0.441–0.902
N-acetyl-beta-D-glucosaminidase		
Creatinine	0.936	0.865–0.973
Osmolality	0.836	0.699–0.927
Specific gravity	0.426	0.185–0.692
EGF		
Creatinine	0.893	0.787–0.958
Osmolality	0.672	0.442–0.855
Specific gravity	0.581	0.313–0.818

*EGF* epidermal growth factor

### Diurnal variations and day-to-day variations of urinary biomarkers

**Fig. 2:**
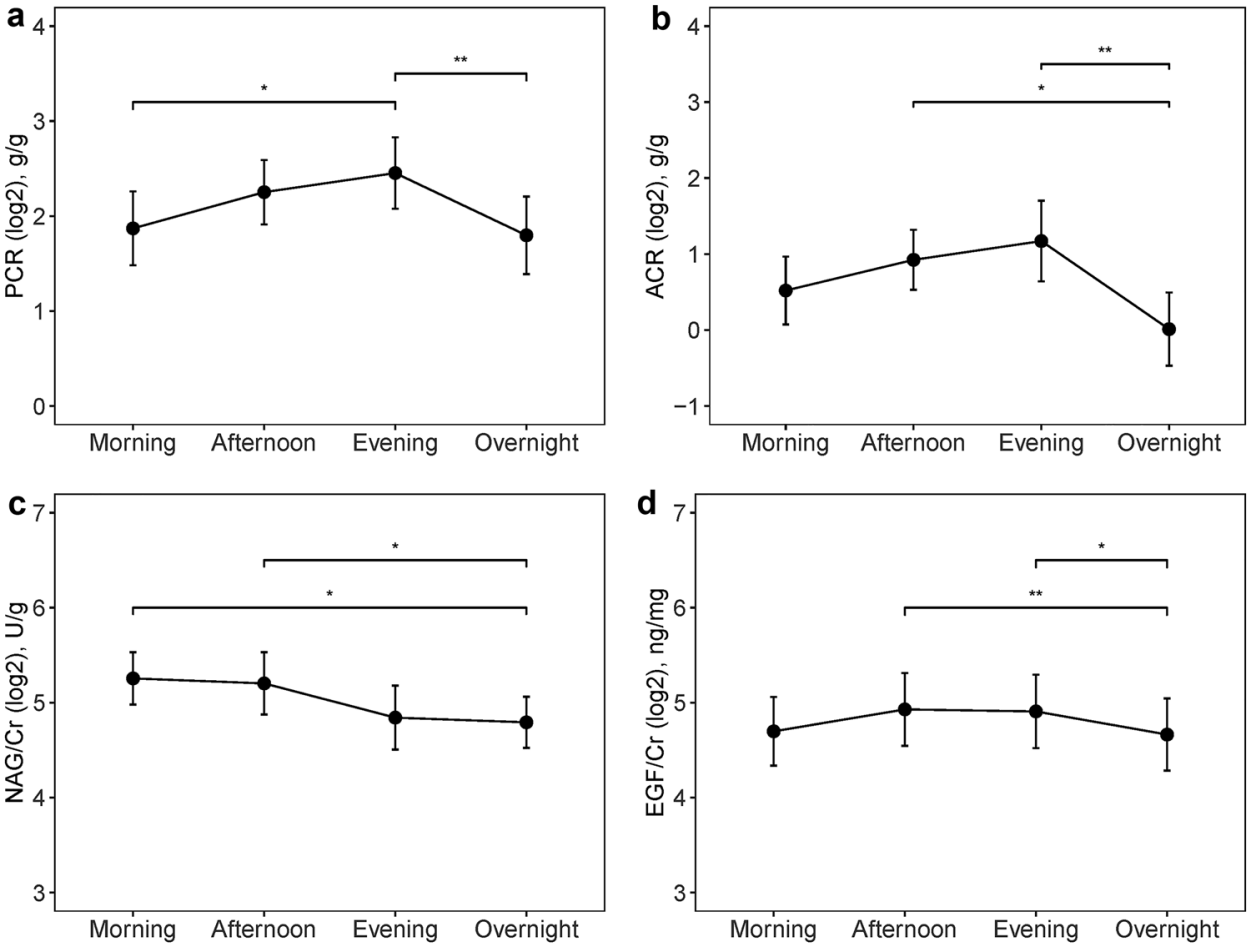
Diurnal variations of the urinary biomarkers within 24 h. Data are described as the mean with standard error. PCR, protein:creatinine ratio; ACR, albumin:creatinine ratio. NAG/Cr, N-acetyl-beta-D-glucosaminidase:creatinine ratio. EGF/Cr, epidermal growth factor:creatinine ratio. **a** The levels of urinary PCR (*n* = 20). **b** The levels of urinary ACR (*n* = 11). **c** The levels of urinary NAG/Cr (*n* = 20). **d** The levels of urinary EGF/Cr (*n* = 17). *p < 0.05. **p < 0.01

As shown in Fig. 2, the levels of PCR, albumin:creatinine ratio (ACR), N-acetyl-beta-D-glucosaminidase:creatinine ratio (NAG/Cr), and EGF:creatinine ratio (EGF/Cr) showed significant diurnal variations across four periods over 24 h (*p* = 0.001, *p* = 0.003, *p* = 0.003, and *p* = 0.003, respectively), with a trough occurring at the overnight period and a peak occurring at the daytime (evening period, evening period, morning period, and afternoon period, respectively). Further pairwise comparisons for PCR showed significant differences between morning and evening (Bonferroni-adjusted *p* < 0.05), as well as between evening and overnight (Bonferroni-adjusted *p* < 0.01). The levels of ACR and EGF/Cr in the overnight period were significantly lower than those in the afternoon (Bonferroni-adjusted *p* < 0.05 and *p* < 0.01, respectively) and evening (Bonferroni-adjusted *p* < 0.01 and *p* < 0.05, respectively). The NAG/Cr in the overnight period was significantly lower than that in the morning and afternoon (Bonferroni-adjusted *p* < 0.05 for both).

Comparisons of diurnal variations and day-to-day variations were further performed (Table 3). No significant difference was observed for PCR. Within-day variations for the levels of ACR and NAG/Cr were significantly higher than their corresponding day-to-day variations. The levels of EGF/Cr exhibited relatively low within-day variations and day-to-day variations (median: 10.2% and 10.6%, respectively).

**Table 3:** Within-day variations and day-to-day variations of urinary biomarkers

	Within-day variation (%)	Day-to-day variation (%)	*p*-value
PCR (g/g, *n* = 20)	25.0 (19.5–37.2)	21.1 (9.7–36.3)	0.409
ACR (mg/g, *n* = 11)	46.0 (33.8–51.7)	21.6 (17.8–29.3)	0.037
NAG/Cr (U/g, *n* = 20)	28.4 (16.6–38.5)	10.9 (5.9–19.2)	0.002
EGF/Cr (ng/mg, *n* = 17)	10.2 (7.4–14.4)	10.6 (5.1–21.0)	0.579

Data are described as the median with interquartile range

*PCR* protein:creatinine ratio, *ACR* albumin:creatinine ratio, *NAG/Cr* N-acetyl-beta-D-glucosaminidase:creatinine ratio, *EGF/Cr* epidermal growth factor:creatinine ratio

### The interaction test between time and other characteristics for biomarker concentrations

Patients were further divided into different subgroups according to age, obesity, usage of diuretics, or blood product transfusion, respectively (Supplementary Figs. 2–4). The results of the linear mixed model showed that adolescent patients had lower levels of PCR (*p* = 0.031) and EGF/Cr (*p* = 0.021), compared with preadolescent patients. Patients in the subgroup with diuretics usage exhibited higher levels of PCR (*p* = 0.003) and NAG/Cr (*p* = 0.002) than those without diuretics usage. Test for interaction showed no significant difference, indicating no group difference in the diurnal pattern of urinary biomarkers (all *p* interaction > 0.05).

### Agreement between each period and 24-h urine samples

As shown in Table 4, the levels of PCR, NAG/Cr, and EGF/Cr in each period showed excellent agreement with their corresponding 24-h levels (*ICC* > 0.9). ACR in the afternoon showed excellent agreement with 24-h levels (*ICC* > 0.9) but moderate or good agreement in the morning, evening, and overnight (*ICC* 0.747–0.848).

**Table 4:** Agreement in the urinary biomarkers between each period and the 24-h urine

	ICC (95% *CI*)	Concentrations normalized by 24-h levels (95% *CI*)
PCR		
Morning	0.971 (0.927–0.989)	0.94 (0.81–1.07)
Afternoon	0.978 (0.945–0.992)	1.08 (0.96–1.21)
Evening	0.928 (0.738–0.975)	1.25 (1.08–1.41)a
Overnight	0.972 (0.926–0.989)	0.84 (0.74–0.94)
ACR		
Morning	0.848 (0.534–0.957)	0.98 (0.71–1.24)
Afternoon	0.923 (0.736–0.98)	1.19 (0.90–1.48)
Evening	0.747 (0.087–0.937)	1.39 (1.09–1.69)a
Overnight	0.796 (0.267–0.945)	0.66 (0.49–0.84)b
NAG/Cr		
Morning	0.979 (0.944–0.992)	1.13 (1.02–1.24)
Afternoon	0.961 (0.859–0.987)	1.19 (1.03–1.35)
Evening	0.981 (0.952–0.993)	0.94 (0.79–1.09)
Overnight	0.978 (0.871–0.993)	0.86 (0.79–0.92)
EGF/Cr		
Morning	0.960 (0.890–0.986)	0.99 (0.90–1.08)
Afternoon	0.945 (0.841–0.981)	1.09 (1.03–1.15)
Evening	0.981 (0.934–0.994)	1.06 (1.01–1.12)
Overnight	0.939 (0.841–0.978)	0.92 (0.86–1.00)

*ICC* intraclass correlation coefficient, *PCR* protein:creatinine ratio, *ACR* albumin: creatinine ratio, *NAG/Cr* N-acetyl-beta-D-glucosaminidase: creatinine ratio, *EGF/Cr* epidermal growth factor: creatinine ratio

^a^Ratio > 1.2

^b^Ratio < 0.8

Furthermore, it could be observed that the urinary PCR and ACR during the evening overestimated 24-h urinary PCR and ACR. Urinary ACR during the overnight period underestimated the level of 24-h urinary ACR. No underestimation or overestimation of 24-h urinary biomarkers was observed in the EGF/Cr levels.

### The impact of processing methods on urinary biomarkers

As shown in Table 5, no significant difference was observed in the levels of urinary EGF/Cr, after urine samples were processed with different methods, including centrifugation, addition of azide sodium and/or protease inhibitors, delayed processing for 96 h, or cryopreservation at a suboptimal temperature of −20 °C.

**Table 5:** The levels of urinary EGF/Cr under different processing and storage conditions

Group	EGF/Cr (ng/mg)	Relative EGF/Cr
Group A	30.8 (17.8–54.5)a	1.01 (0.97–1.11)
Group B	28.1 (18.7–53.7)	Reference
Group C	30.3 (17.6–51.0)a	1.00 (0.96–1.05)
Group D	30.3 (19.93–51.0)a	1.01 (0.95–1.11)
Group E	29.7 (17.4–49.51)a	1.01 (0.94–1.08)
Group F	32.3 (19.9–52.4)a	1.02 (0.89–1.14)
Group G	28.2 (17.6–51.9)a	1.01 (0.96–1.05)

Data are expressed as the median with interquartile range

*EGF/Cr*, epidermal growth factor:creatinine ratio. Group A, timely processing, uncentrifuged urine, no additives, stored at −80 °C. Group B, timely processing, no additives, supernatant, stored at −80 °C. Group C, timely processing, sodium azide only, supernatant, stored at −80 °C. Group D, timely processing, protease inhibitor only, supernatant, stored at −80 °C. Group E, timely processing, sodium azide, and protease inhibitor, supernatant, stored at −80 °C. Group F, timely processing, no additives, supernatant, stored at −20 °C. Group G, delayed processing, supernatant, no additives, stored at −80 °C. a, *p* > 0.05 compared with the urinary biomarker levels of group B, as the reference

## Discussion

The current study investigated the intraindividual variation of urinary biomarkers under different pre-analytical conditions. Because patients’ hydration is well known to dramatically affect the concentration of urinary proteins, we first used three commonly used correction factors to adjust for biomarker concentrations. The 24-h urine collection was split into four distinct periods in terms of the schedule of meals and sleep for hospitalized children in our center. It was observed that the creatinine-normalized concentrations of urinary biomarkers provided the best repeatability across four periods during 24 h, compared with concentrations normalized by osmolality and specific gravity. Thus, we used creatinine-normalized concentrations to conduct the following analysis. There were significant diurnal variations in the concentrations of urinary PCR, ACR, NAG/Cr, and EGF/Cr across four periods over 24 h in pediatric patients with glomerular diseases, with a trough occurring during the overnight period and a peak occurring at a certain period during the daytime. The diurnal patterns of urinary PCR, ACR, and NAG/Cr in children with glomerular diseases were similar with that in adults [34–37] and healthy children [25].

However, few studies compared variations of urinary proteins within a day and between different days. Our results revealed that the variations within a day for urinary ACR and NAG/Cr were significantly higher than their corresponding day-to-day variations. It emphasized the importance of collecting urine samples from the same periods in the daily monitoring of kidney function and urinary biomarkers. Previous researches reported that the biomarker concentrations were associated with age [6], obesity [38], usage of diuretics [39], and blood product transfusion [40], respectively. It is unclear whether the diurnal variations of these four biomarkers would be influenced by these factors. To further explore their potential effect, we performed subgroup analysis by stratifying patients according to age, obesity, diuretic usage, and blood product transfusion, respectively. Test for the interaction showed that the diurnal pattern was not significantly affected by these factors. However, multivariate analysis was not performed due to limited sample size.

Comparisons for biomarker concentrations between each period and 24 h were further performed. The urine samples at the afternoon period overestimated 24-h urinary PCR and ACR. The overnight urine underestimated the 24-h ACR, which is consistent with the results in adults with lupus nephritis [34]. The levels of 24-h urinary biomarkers in this study were calculated based on the weighted average of their corresponding concentrations of four periods considering different volumes of each period. Therefore, the calculated 24-h levels were theoretically equivalent to the actual 24-h levels.

The potential mechanisms underlying the diurnal variations might be associated with the circadian clock [19] and/or some external factors (such as physical activity and food). This may partially explain the diurnal variations of the biomarkers we observed in our study. However, the detailed mechanism needs further investigation. Given the diurnal variations of the above commonly used urinary biomarkers and novel molecular biomarkers, the collection time of the day for urine samples should be recorded, and the impact of time should be taken into account when designing clinical studies and interpreting the results of urinary biomarkers in clinical practice.

Notably, this study investigated the intraindividual variations of EGF/Cr in urine, which has been validated by quite a few researches as a novel promising biomarker associated with kidney outcomes [5–11]. In contrast with the previous research in healthy adults [26, 27], there was significant diurnal variation for urinary EGF/Cr observed by our study. The difference of the results may be associated with the various analytical methods. Our study used the method of ELISA, which had higher sensitivity and specificity than other methods [41]. It allows the variations of EGF/Cr to be more easily detected. Although urinary EGF/Cr during the overnight was significantly lower than that during the afternoon or evening, its variations within a day and between different days were both relatively low (10.2% and 10.6%, respectively), far lower than the variation of 30% reported by a previous study [42]. No overestimation or underestimation was found at any period during 24 h. These results provide novel and valuable evidence to support urinary EGF as a reliable biomarker for future application.

Urinary PCR, ACR, and NAG/Cr have been widely used in clinical practice. They could be tested immediately or within several hours after sample collection. However, urine samples used for EGF/Cr measurement are generally cryopreserved for a period of time until batch test. During the time gap from the sample collection to EGF/Cr measurement, the impact of processing methods and storage conditions remains unclear. Thus, EGF/Cr was included in the investigation for the potential impact of processing methods and storage conditions. We observed that the concentrations of urinary EGF/Cr were not significantly affected by up to 96 h of delayed processing, consistent with a previous study [28]. Moreover, there was no significant impact on the concentrations of urinary EGF either by eliminating the centrifugation step or by the lack of sodium azide and/or protease inhibitor in the urine samples. The levels of EGF can be measured in urine samples collected from clinical centers that are lack of centrifuge or immediate access to −20 °C or −80 °C freezers or in urine samples without preservatives or proteinase inhibitors. The stability of EGF in the urine supports its practical clinical utility, especially in multicenter studies.

Considering the study design of repeated measurements in this study, we only enrolled 20 patients. Previous investigations on intraindividual variations typically involved 10–20 individuals [28, 43]. Four measurements within a day from each patient allow us to obtain sufficient statistical power to detect the diurnal variations and provide clinically useful information. One major strength of this study is that it only included hospitalized children with glomerular diseases. They all had very well-characterized clinical diagnoses. They were given the same schedule of meals and sleep during hospitalization. We have also collected the actual detailed time of patients’ meals and sleep via a paper-based self-reporting questionnaire. Although the questionnaire was not validated, patients or their guardians were requested to fill in the questionnaire before each meal and sleep, respectively, to avoid recall bias. The data showed that patients exhibited similar schedule of meals and sleep. All patients were well educated to avoid strenuous exercise during sample collection. These could reduce the effect of many external factors. Also, the four investigated biomarkers included both commonly used biomarkers and novel molecular biomarkers. They all showed similar diurnal pattern, which provided more information about the diurnal variation of urinary biomarkers in children with kidney diseases. EGF in urine had highly restricted intrarenal expression [44]. So from this point of view, it is superior to some other novel biomarkers (such as kidney injury molecule-1, monocyte chemoattractant protein-1, and YKL-40 [45]) associated with kidney diseases. Thus, EGF was included as a novel biomarker in this study for further investigation of the processing methods and storage conditions.

The weakness of this study is that the effect of the antiproteinuric drugs (angiotensin-converting enzyme inhibitors or angiotensin-receptor blockers, steroids, and nonsteroid immunosuppressive agents) on the diurnal pattern of urinary biomarkers was not clear. It was difficult to determine under the current study design, since most of the patients were prescribed these drugs during hospitalization.

## Conclusions

Compared with osmolality and specific gravity, creatinine-normalized concentrations of urinary biomarkers provided the best repeatability. Given that urinary biomarkers showed significant diurnal variations during 24 h, it is suggested to collect urine from the same period in monitoring their daily changes if possible. The impact of sample collection time should be considered in the interpretation of the results of urinary biomarker measurement. The relatively low variation and excellent agreement with 24-h levels for EGF also extend the evidence for it as a relatively stable biomarker applied in the future clinical practice.

## Supplementary Information

Below is the link to the electronic supplementary material.

Supplementary file1 (PDF 394 KB)

## Data Availability

The data supporting the results of this study are available from the corresponding author upon reasonable request.

## Abbreviations

ACR: Albumin:creatinine ratio
ACEi/ARBs: Angiotensin-converting enzyme inhibitors/angiotensin receptor blockers
BMI: Body mass index
EGF: Epidermal growth factor
EGF/Cr: Epidermal growth factor:creatinine ratio
eGFR: Estimated glomerular filtration rate
ICC: Intraclass correlation coefficient
NAG/Cr: N-Acetyl-beta-D-glucosaminidase:creatinine ratio
PCR: Protein:creatinine ratio
