# Supplementary material for: Intraindividual variations of urinary biomarkers in hospitalized children with glomerular diseases: a prospective observational study
Source: Eur J Pediatr. 2023 Jun 10;182(8):3755–64. doi: 10.1007/s00431-023-05042-9 (PMC10460332; doi:10.1007/s00431-023-05042-9)
Supplement: Supplementary file 1 — Supplementary file1 (PDF 394 KB) [file 431_2023_5042_MOESM1_ESM.pdf]

Intra-individual variations of urinary biomarkers in hospitalized children with glomerular diseases: a prospective observational study

European Journal of Pediatrics

Jianmei Zhou1†, Xuhui Zhong1†, Huijie Xiao1, Ke Xu1, Viji Nair2, Maria Larkina2, Wenjun Ju2*, Jie Ding1*

1 Department of Pediatrics, Peking University First Hospital, Beijing, China

2 Department of Internal Medicine, University of Michigan, Ann Arbor, MI, United States

†These authors share first authorship.

*These authors share senior authorship.

Correspondence:

Wenjun Ju, wenjunj@med.umich.edu; Jie Ding: djnc_5855@126.com.

Supplementary file 1: Questionnaire on the time of meals and sleep for children with glomerular diseases

Dear parents:

This questionnaire is designed by researchers to record the time of children' meals and sleep during the urine collection in the study, which aims to investigate the impact of urine specimen collection time, processing methods, and storage conditions on the levels of biomarkers in children with glomerular diseases. Please fill in the information before each meal and sleep, respectively. The questionnaire will be taken back by the assigned investigator after urine collection was completed. Thank you for your cooperation!

**Name**		**Date**	
**ID**		**Sex**	☐Male ☐Female
**Time for getting up**			
**Time for breakfast**			
**Time for lunch**			
**Time for dinner**			
**Time for going to bed**			

Thanks for your cooperation!

We hope your child will get well soon!

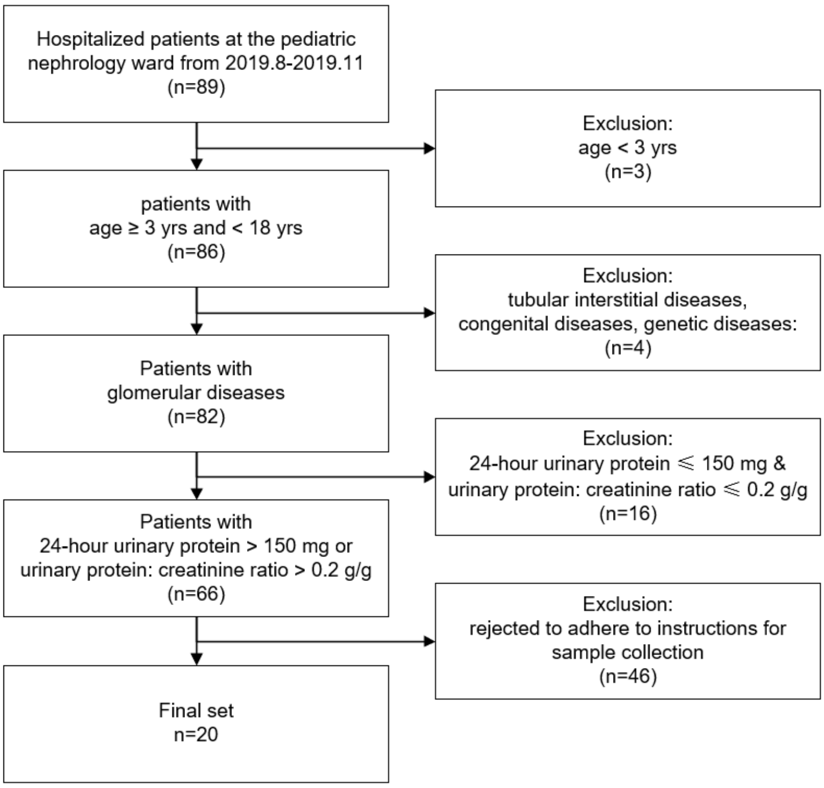

Supplementary Fig. 1 Flow diagram for patient enrollment

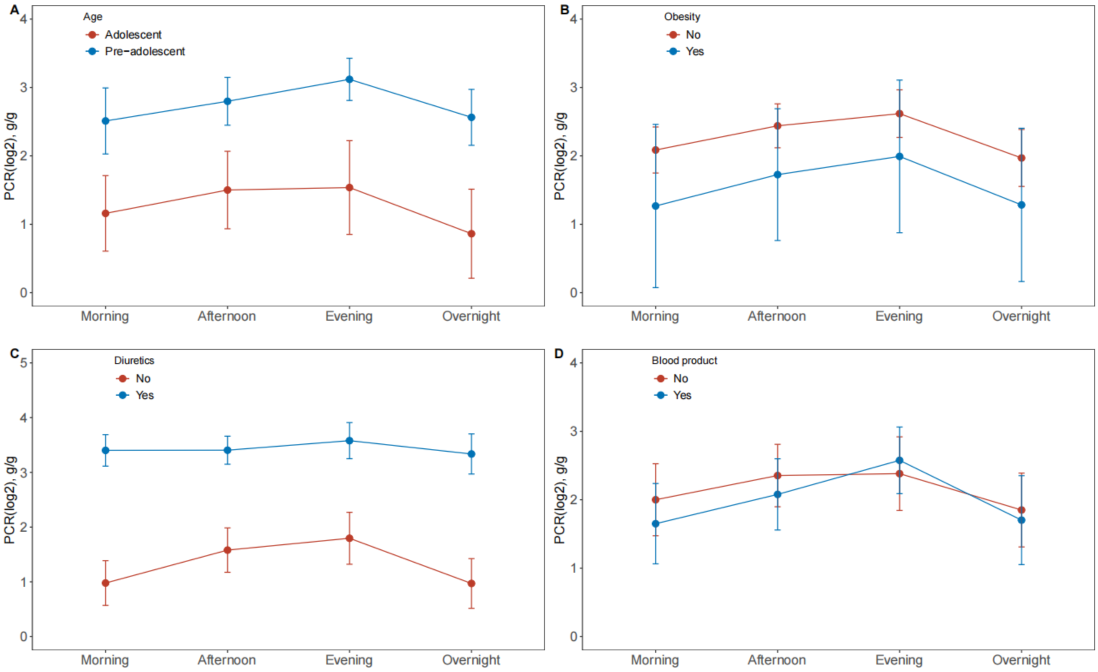

Supplementary Fig. 2 Diurnal variation of PCR after patients were stratified into different subgroups according to age, obesity, diuretics, and blood product transfusion, respectively. Data are described as the mean with standard error. PCR: protein:creatinine ratio.

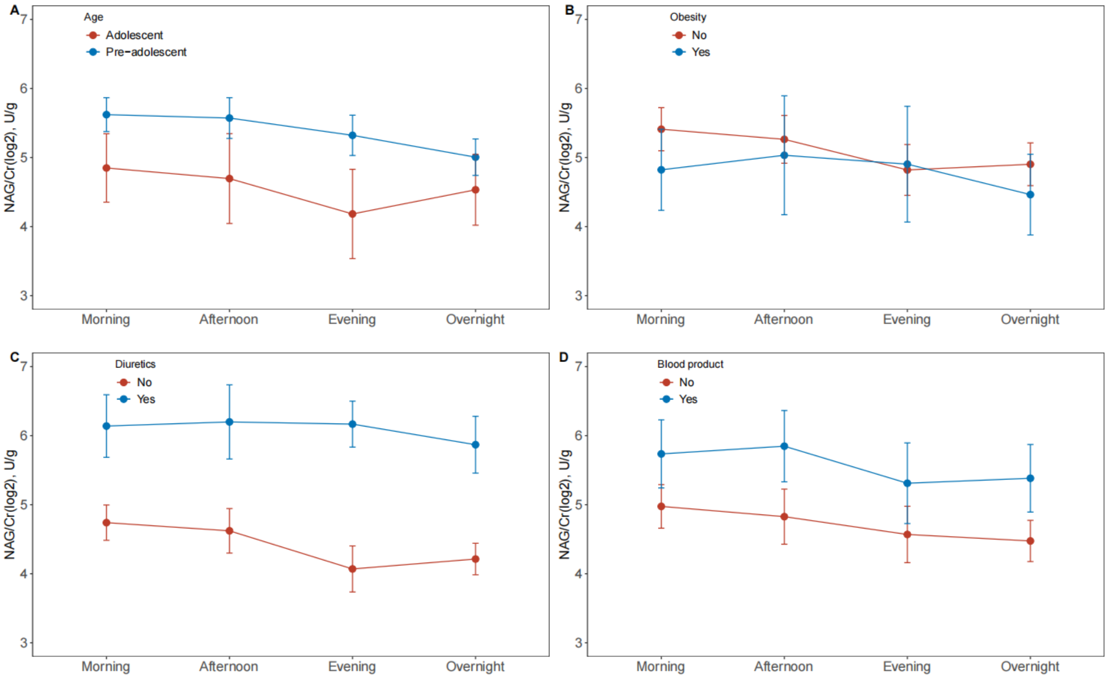

Supplementary Fig. 3 Diurnal variation of NAG/Cr after patients were stratified into different subgroups according to age, obesity, diuretics, and blood product transfusion, respectively. Data are described as the mean with standard error. NAG/Cr: N-acetyl-beta-D-glucosaminidase:creatinine ratio.

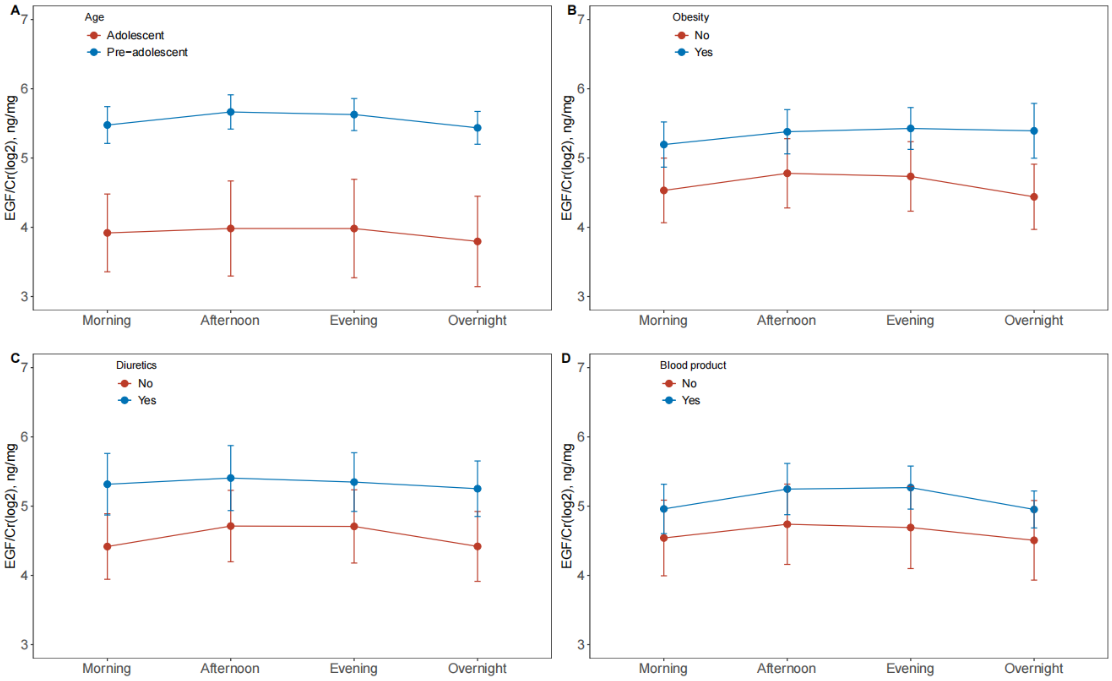

Supplementary Fig. 4 Diurnal variation of EGF/Cr after patients were stratified into different subgroups according to age, obesity, diuretics, and blood product transfusion, respectively. Data are described as the mean with standard error. EGF/Cr: epidermal growth factor: creatinine ratio
